# Some New *q*-Congruences for Truncated Basic Hypergeometric Series: Even Powers

**DOI:** 10.1007/s00025-019-1126-4

**Published:** 2019-11-28

**Authors:** Victor J. W. Guo, Michael J. Schlosser

**Affiliations:** 1 School of Mathematics and Statistics, Huaiyin Normal University, Huai’an, 223300 Jiangsu People’s Republic of China; 2 Fakultät für Mathematik, Universität Wien, Oskar-Morgenstern-Platz 1, 1090 Vienna, Austria

**Keywords:** Basic hypergeometric series, supercongruences, *q*-congruences, cyclotomic polynomial, Andrews’ transformation, Primary 33D15, Secondary 11A07, 11B65

## Abstract

We provide several new *q*-congruences for truncated basic hypergeometric series with the base being an even power of *q*. Our results mainly concern congruences modulo the square or the cube of a cyclotomic polynomial and complement corresponding ones of an earlier paper containing *q*-congruences for truncated basic hypergeometric series with the base being an odd power of *q*. We also give a number of related conjectures including *q*-congruences modulo the fifth power of a cyclotomic polynomial and a congruence for a truncated ordinary hypergeometric series modulo the seventh power of a prime greater than 3.

## Introduction

In his first letter to Hardy from 1913, Ramanujan announced that (cf. [2, p. 25, Equation (2)])

1.1 $$\begin{aligned} \sum _{k=0}^\infty (8k+1)\frac{(\frac{1}{4})_k^4}{k!^4} =\frac{2\sqrt{2}}{\sqrt{\pi }\,\Gamma (\frac{3}{4})^2}, \end{aligned}$$

along with similar hypergeometric identities. Here $(a)_n=a(a+1)\cdots (a+n-1)$ denotes the Pochhammer symbol. He did not provide proofs. This identity was eventually proved by Hardy in [19, p. 495].

In 1997, Van Hamme [32] proposed 13 interesting *p*-adic analogues ofRamanujan-type formulas for $1/\pi$ [26], such as

1.2 $$\begin{aligned} \sum _{k=0}^{(p-1)/4}(8k+1)\frac{(\frac{1}{4})_k^4}{k!^4} \equiv p\frac{\Gamma _p(\frac{1}{2})\Gamma _p(\frac{1}{4})}{\Gamma _p(\frac{3}{4})} \pmod {p^3},\quad \text {if }p\equiv 1\pmod {4}, \end{aligned}$$

where *p* is an odd prime and $\Gamma _p$ is the *p*-adic gamma function [22]. Van Hamme [32] himself proved three of them. Nowadays all of the 13 supercongruences have been confirmed by different techniques (see [20, 21, 23, 25, 30]). For some informative background on Ramanujan-type supercongruences, we refer the reader to Zudilin’s paper [35]. During the past few years, congruences and supercongruences have been generalized to the *q*-world by many authors (see, for example, [6–18, 24, 27, 31]). As explained in [18], *q*-supercongruences are closely related to studying the asymptotic behaviour of *q*-series at roots of unity.

Recently, the authors [15, Theorems 1 and 2] proved that for odd $d\geqslant 5$,

1.3 $$\begin{aligned} \sum _{k=0}^{n-1}[2dk+1]\frac{(q;q^d)_k^d}{(q^d;q^d)_k^d}q^{\frac{d(d-3)k}{2}} \equiv {\left\{ \begin{array}{ll} 0\pmod {\Phi _n(q)^2}, &{}\text {if }n\equiv -1\pmod {d},\\ 0\pmod {\Phi _n(q)^3}, &{}\text {if }n\equiv -\frac{1}{2}\pmod {d}, \end{array}\right. } \end{aligned}$$

and for odd $d\geqslant 3$ and $n>1$,

1.4 $$\begin{aligned} \sum _{k=0}^{n-1}[2dk-1]\frac{(q^{-1};q^d)_k^d}{(q^d;q^d)_k^d} q^{\frac{d(d-1)k}{2}} \equiv {\left\{ \begin{array}{ll} 0\pmod {\Phi _n(q)^2}, &{}\text {if }n\equiv 1\pmod {d},\\ 0\pmod {\Phi _n(q)^3}, &{}\text {if }n\equiv \frac{1}{2}\pmod {d}. \end{array}\right. } \end{aligned}$$

Here and throughout the paper, we adopt the standard *q*-notation: For an indeterminate *q*, let

$$\begin{aligned} (a;q)_n=(1-a)(1-aq)\cdots (1-aq^{n-1}) \end{aligned}$$

be the *q-shifted factorial*. For convenience, we compactly write

$$\begin{aligned} (a_1,a_2,\ldots ,a_m;q)_n=(a_1;q)_n (a_2;q)_n\cdots (a_m;q)_n \end{aligned}$$

for a product of *q*-shifted factorials. Moreover,

$$\begin{aligned}{}[n]=[n]_q=1+q+\cdots +q^{n-1} \end{aligned}$$

denotes the *q*-*integer*, which can be defined by $[n]=(q^n-1)/(q-1)$ to hold for any integer *n*, including negative *n*, which in particular gives $[-1]=-1/q$ (which is needed in the $k=0$ terms of (1.6) and (1.8) and at other places in this paper). Furthermore, $\Phi _n(q)$ denotes the *n*th *cyclotomic polynomial* in *q*, which may be defined as

$$\begin{aligned} \Phi _n(q)=\prod _{\begin{array}{c} 1\leqslant k\leqslant n\\ \gcd (n,k)=1 \end{array}}(q-\zeta ^k), \end{aligned}$$

where $\zeta$ is an *n*th primitive root of unity.

In this paper, we shall prove results similar to (1.3) and (1.4) for *even* *d*. The first result concerns the case $d=2$.

### Theorem 1

Let *n* be an odd integer greater than 1. Then

1.5 $$\begin{aligned} \sum _{k=0}^{n-1}[4k+1]\frac{(q;q^2)_k^2}{(q^2;q^2)_k^2} q^{-k}&\equiv q[n]^2\pmod {[n]^2\Phi _n(q)}, \end{aligned}$$

1.6 $$\begin{aligned} \sum _{k=0}^{n-1}[4k-1]\frac{(q^{-1};q^2)_k^2}{(q^2;q^2)_k^2} q^{k}&\equiv -[n]^2\pmod {[n]^2\Phi _n(q)}. \end{aligned}$$

### Theorem 2

Let $d\geqslant 4$ be an even integer and let *n* be a positive integer with $n\equiv -1\pmod {d}$. Then

1.7 $$\begin{aligned} \sum _{k=0}^{n-1}[2dk+1]\frac{(q;q^d)_k^d}{(q^d;q^d)_k^d}q^{\frac{d(d-3)k}{2}} \equiv 0\pmod {\Phi _n(q)^2}. \end{aligned}$$

### Theorem 3

Let $d\geqslant 4$ be an even integer and let $n>1$ be an integer with $n\equiv 1\pmod {d}$. Then

1.8 $$\begin{aligned} \sum _{k=0}^{n-1}[2dk-1]\frac{(q^{-1};q^d)_k^d}{(q^d;q^d)_k^d}q^{\frac{d(d-1)k}{2}} \equiv 0\pmod {\Phi _n(q)^2}. \end{aligned}$$

Although neither (1.7) nor (1.8) holds modulo $\Phi _n(q)^3$ in general, we have the following common refinement of (1.3) and (1.7).

### Theorem 4

Let $d\geqslant 4$ be an integer and let *n* be a positive integer with $n\equiv -1\pmod {d}$. Then

1.9 $$\begin{aligned} \sum _{k=0}^{n-1}[2dk+1]\frac{(q;q^d)_k^d}{(q^d;q^d)_k^d}q^{\frac{d(d-3)k}{2}} \equiv 0\pmod {\Phi _n(q)^2\Phi _{dn-n}(q)}. \end{aligned}$$

Let $n=p$ be an odd prime and $d=p+1$ in (1.9). Then letting $q\rightarrow 1$, we are led to

1.10 $$\begin{aligned} \sum _{k=0}^{p-1}(2p+2k+1)\frac{(\frac{1}{p+1})_k^{p+1}}{k!^{p+1}} \equiv 0\pmod {p^3}. \end{aligned}$$

Note that Sun [28, Theorem 1.2] proved that for any prime $p>3$

1.11 $$\begin{aligned} \sum _{k=0}^{p-1}\frac{(\frac{1}{p+1})_k^{p+1}}{k!^{p+1}} \equiv 0\pmod {p^5}, \end{aligned}$$

which also holds modulo $p^3$ for $p=3$. Substituting (1.11) into (1.10), we arrive at the following conclusion.

### Corollary 5

Let *p* be an odd prime. Then

$$\begin{aligned} \sum _{k=0}^{p-1}k\frac{(\frac{1}{p+1})_k^{p+1}}{k!^{p+1}}\equiv 0\pmod {p^3}. \end{aligned}$$

This result is actually a special case of

1.12 $$\begin{aligned} \sum _{k=0}^{p-1}k\frac{(\frac{1}{p+1})_k^{p+1}}{k!^{p+1}}\equiv \frac{p^3}{4}-\frac{p^4}{8}\pmod {p^5}, \end{aligned}$$

that was conjectured by Sun and was subsequently proved by Gao [5] in her master thesis. See the discussion around Equation (1.3) in Wang’s paper [33] where (1.12) is further generalized to a congruence modolo $p^6$ for $p>5$ that involves Bernoulli numbers. In Sect. 5 we propose an extension of Corollary 5 which contains additional factors in the summand (see Conjecture 1).

The paper is organized as follows. We shall prove Theorem 1 in Sect. 2 based on two *q*-series identities. Theorems 2 and 3 will be proved by giving a common generalization of them in Sect. 3. To accomplish this we shall make a careful use of Andrews’ multiseries generalization of the Watson transformation [1, Theorem 4] (which was already used in [12] to prove some *q*-analogues of Calkin’s congruence [3], and which was also applied in [15] for proving some analogous results involving the base being odd powers of *q*). We shall prove Theorem 4 by using a certain anti-symmetry of the *k*-th summand on the left-hand side of (1.9) in Sect. 4. Finally, in Sect. 5 we give some concluding remarks and state some open problems. These include some conjectural *q*-congruences modulo the fifth power of a cyclotomic polynomial and congruences for truncated ordinary hypergeometric series, one of them, see (5.7), modulo the seventh power of a prime greater than 3.

We would like to thank the two anonymous referees for their comments. We especially thank the second referee for her or his detailed list of constructive suggestions for improvement of the paper.

## Proof of Theorem 1

It is easy to prove by induction on *n* that

$$\begin{aligned} \sum _{k=0}^{n-1}[4k+1]\frac{(q;q^2)_k^2}{(q^2;q^2)_k^2} q^{-k} =[n]^2(1+q^n)^2\frac{(q;q^2)_n^2}{(q^2;q^2)_n^2}q^{1-n}&\\ =[n]^2\begin{bmatrix}2n-1\\n-1\end{bmatrix}^2\frac{q^{1-n}}{(-q;q)_{n-1}^4}&. \end{aligned}$$

Since $q^n\equiv 1\pmod {\Phi _n(q)}$, the proof of (1.5) then follows from the fact

$$\begin{aligned} \begin{bmatrix}2n-1\\n-1\end{bmatrix}= \prod _{k=1}^{n-1}\frac{1-q^{2n-k}}{1-q^k} \equiv q^{-\left( {\begin{array}{c}n\\ 2\end{array}}\right) }(-1)^{n-1}\equiv 1\pmod {\Phi _n(q)} \end{aligned}$$

for odd *n* and $(-q;q)_{n-1}\equiv 1\pmod {\Phi _n(q)}$ (see, for example, [8, Equation (2.3)]).

Similarly, we can prove by induction that

$$\begin{aligned} \sum _{k=0}^{n-1}[4k-1]\frac{(q^{-1};q^2)_k^2}{(q^2;q^2)_k^2} q^{k} =-[n]^2(1+q^n)^2\frac{(q^{-1};q^2)_n^2}{(q^2;q^2)_n^2}q^{n}. \end{aligned}$$

The proof of (1.6) then follows from that of (1.5) and the following relation

$$\begin{aligned} (q^{-1};q^2)_n=(q;q^2)_{n}\frac{1-q^{-1}}{1-q^{2n-1}}. \end{aligned}$$

We point out that, using the congruence $(-q;q)_{(n-1)/2}^2\equiv q^{(n^2-1)/8}\pmod {\Phi _n(q)}$ for odd *n* (see, for example, [8, Lemma 2.1]), we can prove the following similar congruences: for any odd positive integer $n>1$,

2.1 $$\begin{aligned} \sum _{k=0}^{(n-1)/2}[4k+1]\frac{(q;q^2)_k^2}{(q^2;q^2)_k^2} q^{-k}&\equiv q[n]^2\pmod {[n]^2\Phi _n(q)}, \end{aligned}$$

2.2 $$\begin{aligned} \sum _{k=0}^{(n+1)/2}[4k-1]\frac{(q^{-1};q^2)_k^2}{(q^2;q^2)_k^2} q^{k}&\equiv -[n]^2\pmod {[n]^2\Phi _n(q)}. \end{aligned}$$

The details of the proof are left to the interested reader.

## Proof of Theorems 2 and 3

We shall first prove the following unified generalization of Theorems 2 and 3 for $d=4$.

### Theorem 6

Let *r* be an odd integer. Let $n>1$ be an odd integer with $n\equiv -r\pmod {4}$ and $n\geqslant \max \{r,4-r\}$. Then

3.1 $$\begin{aligned} \sum _{k=0}^{n-1}[8k+r]\frac{(q^r;q^4)_k^4}{(q^4;q^4)_k^4}q^{(4-2r)k} \equiv 0\pmod {\Phi _n(q)^2}. \end{aligned}$$

### Proof

Let $\alpha$, *j* and *r* be integers. It is easy to see that

$$\begin{aligned} (1-q^{\alpha n-dj+d-r})(1-q^{\alpha n+dj-d+r})+(1-q^{dj-d+r})^2 q^{\alpha n-dj+d-r}=(1-q^{\alpha n})^2 \end{aligned}$$

and $1-q^{\alpha n}\equiv 0\pmod {\Phi _n(q)}$, and so

$$\begin{aligned} (1-q^{\alpha n-dj+d-r})(1-q^{\alpha n+dj-d+r})\equiv -(1-q^{dj-d+r})^2 q^{\alpha n-dj+d-r}\!\!\!\pmod {\Phi _n(q)^2}. \end{aligned}$$

It follows that

3.2 $$\begin{aligned} (q^{r-\alpha n},q^{r+\alpha n};q^d)_k \equiv (q^r;q^d)_k^2 \pmod {\Phi _n(q)^2}. \end{aligned}$$

It is clear that $3n\equiv r\pmod {4}$. Therefore, by (3.2) and the $q\mapsto q^4$, $a\mapsto q^r$, $b\mapsto q^r$, $c\mapsto q^{r+3n}$, $n\mapsto (3n-r)/4$ instance of the terminating ${}_6\phi _5$ summation (see [4, Appendix (II.21)]):

$$\begin{aligned} _6\phi _5 \left[ \begin{array}{c} a,\, qa^{\frac{1}{2}},\, -qa^{\frac{1}{2}},\, b,\, c,\, q^{-n} \\ a^{\frac{1}{2}},\, -a^{\frac{1}{2}},\, aq/b,\, aq/c,\, aq^{n+1}\end{array}; q,\,\frac{aq^{n+1}}{bc} \right] =\frac{(aq, aq/bc;q)_n}{(aq/b, aq/c;q)_n}, \end{aligned}$$

where the *basic hypergeometric series *$_{r+1}\phi _r$ (see [4]) is defined as

$$\begin{aligned} _{r+1}\phi _{r}\left[ \begin{array}{c} a_1,a_2,\ldots ,a_{r+1}\\ b_1,b_2,\ldots ,b_{r} \end{array};q,\, z \right] =\sum _{k=0}^{\infty }\frac{(a_1,a_2,\ldots , a_{r+1};q)_k z^k}{(q,b_1,\ldots ,b_{r};q)_k}, \end{aligned}$$

modulo $\Phi _n(q)^2$, the left-hand side of (3.1) is congruent to

3.3 $$\begin{aligned}&\sum _{k=0}^{(3n-r)/4}[8k+r]\frac{(q^r,q^r,q^{r+3n}, q^{r-3n};q^4)_k }{(q^4, q^4, q^{4-3n}, q^{4+3n};q^4)_k} q^{(4-2r)k}&\nonumber \\&\qquad =[r]\frac{(q^{r+4}, q^{4-3n-r};q^4)_{(3n-r)/4}}{(q^4, q^{4-3n};q^4)_{(3n-r)/4}}. \end{aligned}$$

Note that $(3n-r)/4\leqslant n-1$ by the condition $n\geqslant 4-r$. It is clear that $(q^{r+4};q^4)_{(3n-r)/4}$ has the factor $1-q^{3n}$, and $(q^{4-r-3n};q^4)_{(3n-r)/4}$ has the factor $(1-q^{-2n})$ since $(3n-r)/4\geqslant (n+r)/4$ by the condition $n\geqslant r$. Therefore the numerator on the right-hand side of (3.3) is divisible by $\Phi _n(q)^2$, while the denominator is relatively prime to $\Phi _n(q)$. This completes the proof. $\square$

We need the following lemma in our proof of Theorems 2 and 3 for $d\geqslant 6$.

### Lemma 1

Let $d\geqslant 5$ be an integer and let *r* be an integer with $\gcd (d,r)=1$. Let $n=ad-r\geqslant r$ with $a\geqslant 1$. Suppose that $2r+kd\equiv 0\pmod {n}$ for some $k>0$. Then $k\geqslant a(d-4)/2$.

### Proof

Since $\gcd (d,r)=1$, we have $\gcd (n,d)=1$ for $n=ad-r$. Noticing that

$$\begin{aligned} 2r+kd=(k+2a)d-2(ad-r)=(k+2a)d-2n, \end{aligned}$$

we conclude that $(k+2a)d\equiv 0\pmod {n}$. It follows that $k+2a$ is a multiple of *n* and so $k+2a\geqslant n$, i.e.,

$$\begin{aligned} k+2a\geqslant ad-r. \end{aligned}$$

By the condition $ad-r\geqslant r$ in the lemma, we get $r\leqslant ad/2$. Substituting this into the above inequality, we obtain the desired result. $\square$

We now give a common generalization of Theorems 2 and 3.

### Theorem 7

Let $d\geqslant 4$ be an even integer and let *r* be an integer with $\gcd (d,r)=1$. Let $n>1$ be an integer with $n\equiv -r\pmod {d}$ and $n\geqslant \max \{r,d-r\}$. Then

3.4 $$\begin{aligned} \sum _{k=0}^{n-1}[2dk+r]\frac{(q^r;q^d)_k^d}{(q^d;q^d)_k^d}q^{\frac{d(d-r-2)k}{2}} \equiv 0\pmod {\Phi _n(q)^2}. \end{aligned}$$

### Proof

The $d=4$ case is just Theorem 6. We now suppose that $d\geqslant 6$. The proof of this case is intrinsically the same as that of Theorem 6. Here we need to use a complicated transformation formula due to Andrews [1, Theorem 4]:

3.5 $$\begin{aligned}&\sum _{k\geqslant 0}\frac{(a,q\sqrt{a},-q\sqrt{a},b_1,c_1,\dots ,b_m,c_m,q^{-N};q)_k}{(q,\sqrt{a},-\sqrt{a},aq/b_1,aq/c_1,\dots ,aq/b_m,aq/c_m,aq^{N+1};q)_k}\nonumber \\&\qquad \times \left( \frac{a^mq^{m+N}}{b_1c_1\cdots b_mc_m}\right) ^k \nonumber \\&\quad =\frac{(aq,aq/b_mc_m;q)_N}{(aq/b_m,aq/c_m;q)_N} \sum _{l_1,\dots ,l_{m-1}\geqslant 0} \frac{(aq/b_1c_1;q)_{l_1}\cdots (aq/b_{m-1}c_{m-1};q)_{l_{m-1}}}{(q;q)_{l_1}\cdots (q;q)_{l_{m-1}}} \nonumber \\&\quad \quad \times \frac{(b_2,c_2;q)_{l_1}\dots (b_m,c_m;q)_{l_1+\dots +l_{m-1}}}{(aq/b_1,aq/c_1;q)_{l_1} \dots (aq/b_{m-1},aq/c_{m-1};q)_{l_1+\dots +l_{m-1}}} \nonumber \\&\quad \quad \times \frac{(q^{-N};q)_{l_1+\dots +l_{m-1}}}{(b_mc_mq^{-N}/a;q)_{l_1+\dots +l_{m-1}}} \frac{(aq)^{l_{m-2}+\dots +(m-2)l_1} q^{l_1+\dots +l_{m-1}}}{(b_2c_2)^{l_1}\cdots (b_{m-1}c_{m-1})^{l_1+\dots +l_{m-2}}}, \end{aligned}$$

which is a multiseries generalization of Watson’s $_8\phi _7$ transformation formula (see [4, Appendix (III.18)]):

3.6 $$\begin{aligned}&_{8}\phi _{7}\!\left[ \begin{array}{cccccccc} a,&{} qa^{\frac{1}{2}},&{} -qa^{\frac{1}{2}}, &{} b, &{} c, &{} d, &{} e, &{} q^{-n} \\ &{} a^{\frac{1}{2}}, &{} -a^{\frac{1}{2}}, &{} aq/b, &{} aq/c, &{} aq/d, &{} aq/e, &{} aq^{n+1} \end{array};q,\, \frac{a^2q^{n+2}}{bcde} \right] \nonumber \\&\quad =\frac{(aq, aq/de;q)_n}{(aq/d, aq/e;q)_n} \,{}_{4}\phi _{3}\!\left[ \begin{array}{c} aq/bc,\ d,\ e,\ q^{-n} \\ aq/b,\, aq/c,\, deq^{-n}/a \end{array};q,\, q \right] . \end{aligned}$$

It is easy to see that $(d-1)n\equiv r\pmod {d}$. Hence, by (3.2), modulo $\Phi _n(q)^2$, the left-hand side of (3.4) is congruent to

$$\begin{aligned} \sum _{k=0}^{(dn-n-r)/d}&[r]\frac{(q^r,q^d\sqrt{q^r},-q^d\sqrt{q^r}, \overbrace{q^r,\ldots ,q^r}^{{(d-3)\text {'s} q^r}};q^d)_k}{(q^d,\sqrt{q^r},-\sqrt{q^r},q^d,\ldots ,q^d;q^d)_k}\\&\times \frac{(q^{r+(d-1)n},q^{r-(d-1)n};q^d)_k}{(q^{d-(d-1)n},q^{d+(d-1)n};q^d)_k} q^{\frac{d(d-r-2)k}{2}}, \end{aligned}$$

where we have used the fact $(dn-n-r)/d\leqslant n-1$ by the condition $n\geqslant d-r$. Furthermore, by the $q\mapsto q^d$, $a\mapsto q^r$, $b_i\mapsto q^r$, $c_i\mapsto q^r$, for $1\leqslant i\leqslant m-1$, $b_m\mapsto q^r$, $c_m\mapsto q^{r+(d-1)n}$, $N\mapsto ((d-1)n-r)/d$ case of Andrews’ transformation (3.5), the above summation can be written as

3.7 $$\begin{aligned}&[r]\frac{(q^{d+r},q^{d+n-dn-r};q^d)_{(dn-n-r)/d}}{(q^d,q^{d+n-dn};q^d)_{(dn-n-r)/d}} \sum _{l_1,\dots ,l_{m-1}\geqslant 0} \frac{(q^{d-r};q^d)_{l_1}\cdots (q^{d-r};q^d)_{l_{m-1}}}{(q^d;q^d)_{l_1}\cdots (q^d;q^d)_{l_{m-1}}} \nonumber \\&\quad \quad \times \frac{(q^r,q^r;q^d)_{l_1}\dots (q^r,q^{r+(d-1)n};q^d)_{l_1+\dots +l_{m-1}}}{(q^d,q^d;q^d)_{l_1} \dots (q^d,q^d;q^d)_{l_1+\dots +l_{m-1}}} \nonumber \\&\quad \quad \times \frac{(q^{r-(d-1)n};q^d)_{l_1+\dots +l_{m-1}}}{(q^{2r};q^d)_{l_1+\dots +l_{m-1}}} \frac{q^{(d+r)(l_{m-2}+\dots +(m-2)l_1)} q^{d(l_1+\dots +l_{m-1})}}{q^{2rl_1}\cdots q^{2r(l_1+\dots +l_{m-2})}},\nonumber \\ \end{aligned}$$

where $$m=(d-2)/2$$.

It is easy to see that $(q^{d+r};q^d)_{(dn-n-r)/d}$ contains the factor $1-q^{(d-1)n}$. Similarly, $(q^{d+n-dn-r};q^d)_{(dn-n-r)/d}$ contains the factor $1-q^{(2-d)n}$ since $(dn-n-r)/d\geqslant (n+r)/d$ by the conditions $d\geqslant 6$ and $n\geqslant r$. Thus, the expression $(q^{d+r},q^{d+n-dn-r};q^d)_{(dn-n-r)/d}$ in the fraction before the multiple summation is divisible by $\Phi _n(q)^2$.

Note that the non-zero terms in the multiple summation of (3.7) are just those indexed by $(l_1,\ldots ,l_{m-1})$ with $l_1+\dots +l_{m-1}\leqslant (dn-n-r)/d\leqslant n-1$ because of the factor $(q^{r-(d-1)n};q^d)_{l_1+\dots +l_{m-1}}$ in the numerator. This immediately implies that all the other *q*-factorials in the denominator of the multiple summation of (3.7) do not contain factors of the form $1-q^{\alpha n}$ (and are therefore relatively prime to $\Phi _n(q)$), except for $(q^{2r};q^d)_{l_1+\dots +l_{m-1}}$. If $n=d-r$, then it is clear that at least one $(q^{d-r};q^d)_{l_i}$ contains the factor $1-q^n$ $l_1+\dots +l_{m-1}>0$. We now assume that $n\geqslant 2d-r$ and so $n>\max \{d,r\}$ in this case. Thus, if $(q^{2r};q^d)_{l_1+\dots +l_{dm-1}}$ has a factor $1-q^{kn}$, then the number *k* is unique since $l_1+\dots +l_{m-1}\leqslant n-1$ and $\gcd (n,d)=1$. Moreover, if such a *k* exists, then we must have $k\geqslant a(d-4)/2$ by Lemma 1, where $n=ad-r$. It follows that $l_1+\dots +l_{m-1}\geqslant k$ and at least one $l_i$ is greater than or equal to $k/(m-1)=2k/(d-4)\geqslant a$ and so $(q^{d-r};q^d)_{l_i}$ contains the factor $1-q^n$ in this case. This proves that the denominator of the reduced form of the multiple summation of (3.7) is always relatively prime to $\Phi _n(q)$, which completes the proof of (3.4). $\square$

## Proof of Theorem 4

We shall prove

$$\begin{aligned} \sum _{k=0}^{n-1}[2dk+1]\frac{(q;q^d)_k^d}{(q^d;q^d)_k^d}q^{\frac{d(d-3)k}{2}} \equiv 0\pmod {\Phi _{dn-n}(q)}, \end{aligned}$$

which is equivalent to

4.1 $$\begin{aligned} \sum _{k=0}^{(dn-n-1)/d}[2dk+1]\frac{(q;q^d)_k^d}{(q^d;q^d)_k^d}q^{\frac{d(d-3)k}{2}} \equiv 0\pmod {\Phi _{dn-n}(q)}, \end{aligned}$$

because $(q;q^d)_k$ has the factor $1-q^{dn-n}$ and is therefore divisible by $\Phi _{dn-n}(q)$ for $(dn-n-1)/d<k\leqslant n-1$, while $(q^d;q^d)_k$ is coprime with $\Phi _{dn-n}(q)$ for these *k*.

Since $q^{dn-n}\equiv 1\pmod {\Phi _{dn-n}(q)}$, we have

4.2 $$\begin{aligned} \frac{(q;q^d)_{(dn-n-1)/d} }{(q^d;q^d)_{(dn-n-1)/d}}&=\frac{(1-q)(1-q^{d+1})\cdots (1-q^{dn-n-d})}{(1-q^{d})(1-q^{2d})\cdots (1-q^{dn-n-1})} \nonumber \\&\equiv \frac{(1-q)(1-q^{d+1})\cdots (1-q^{dn-n-d})}{(1-q^{-(dn-n-d)})(1-q^{-(dn-n-2d)})\cdots (1-q^{-1})}\nonumber \\&=(-1)^{\frac{dn-n-1}{d}}q^{\frac{(d-1)(n-1)(dn-n-1)}{2d}} \pmod {\Phi _{dn-n}(q)}. \end{aligned}$$

Furthermore, for $0\leqslant k\leqslant (dn-n-1)/d$, we have

$$\begin{aligned}&\frac{(q;q^d)_{(dn-n-1)/d-k} }{(q^d;q^d)_{(dn-n-1)/d-k}}\\&\quad =\frac{(q;q^d)_{(dn-n-1)/d} }{(q^d;q^d)_{(dn-n-1)/d}}\\&\qquad \;\times \frac{(1-q^{dn-n-1-(k-1)d})(1-q^{dn-n-1-(k-2)d}) \cdots (1-q^{dn-n-1})}{(1-q^{dn-n-kd})(1-q^{dn-n-(k-1)d})\cdots (1-q^{dn-n-d})} \\&\quad \equiv (-1)^{\frac{dn-n-1}{d}}q^{\frac{(d-1)(n-1)(dn-n-1)}{2d}}\\&\qquad \;\times \frac{(1-q^{-1-(k-1)d})(1-q^{-1-(k-2)d})\cdots (1-q^{-1})}{(1-q^{-kd})(1-q^{-(k-1)d})\cdots (1-q^{-d})} \\&\quad =(-1)^{\frac{dn-n-1}{d}}q^{\frac{(d-1)(n-1)(dn-n-1)}{2d}+(d-1)k} \frac{(q;q^d)_{k} }{(q^d;q^d)_{k}} \pmod {\Phi _{dn-n}(q)}. \end{aligned}$$

Taking the most left- and right-hand sides of this congruence to the power *d*, it follows, using $q^{dn-n}\equiv 1\pmod {\Phi _{dn-n}(q)}$, that for $0\leqslant k\leqslant (dn-n-1)/d$ there holds

$$\begin{aligned}&[2d((dn-n-1)/d-k)+1]\frac{(q;q^d)_{(dn-n-1)/d-k}^d}{(q^d;q^d)_{(dn-n-1)/d-k}^d}q^{\frac{d(d-3)((dn-n-1)/d-k)}{2}}\\&\quad \equiv (-1)^{dn-n}q^{\frac{(dn-n)(dn-n-3)}{2}} [2dk+1] \frac{(q;q^d)_k^d}{(q^d;q^d)_k^d}q^{\frac{d(d-3)k}{2}} \pmod {\Phi _{dn-n}(q)}. \end{aligned}$$

It is easy to check that $(-1)^{dn-n}q^{\frac{(dn-n)(dn-n-3)}{2}}\equiv -1\pmod {\Phi _{dn-n}(q)}$ whenever $dn-n$ is odd or even. This proves that the *k*-th and $((dn-n-1)/d-k)$-th terms of the left-hand side of (4.1) cancel each other modulo $\Phi _{dn-n}(q)$ and therefore (4.1) holds. Equivalently, (1.9) holds modulo $\Phi _{dn-n}(q)$. Moreover, by (1.3) and (1.7), one sees that (1.9) also holds modulo $\Phi _{n}(q)^2$ for $d\geqslant 4$. The proof then follows from the fact $\Phi _{n}(q)^2$ and $\Phi _{dn-n}(q)$ are relatively prime polynomials.

## Concluding Remarks and Open Problems

Having establishing (*q*-)congruences for truncated (basic) hypergeometric series, one can wonder what their ‘archimedian’ analogues are, i.e. whether the infinite sums from $k=0$ to $\infty$ have known evaluations, just as (1.1) is such an archimedian analogue for (1.2).

In many cases of our results, especially when dealing with arbitrary exponents *d*, we are not aware of explicit evaluations in the archimedian case. However for small *d* we can easily find corresponding evaluations by suitably specializing known summations for (basic) hypergeometric series, such as Rogers’ nonterminating $_6\phi _5$ summation (cf. [4, Appendix (II.20)]),

5.1 $$\begin{aligned}&{}_{6}\phi _5\!\left[ \begin{array}{cccccc} a,&{} qa^{\frac{1}{2}},&{} -qa^{\frac{1}{2}}, &{} b, &{} c, &{} d \\ &{} a^{\frac{1}{2}}, &{} -a^{\frac{1}{2}}, &{} aq/b, &{} aq/c, &{} aq/d \end{array};q,\, \frac{aq}{bcd} \right]&\nonumber \\&\quad =\frac{(aq, aq/bc,aq/bd,aq/cd;q)_\infty }{(aq/b,aq/c,aq/d,aq/bcd;q)_\infty }, \end{aligned}$$

where $|aq/bcd|<1$ for convergence.

Indeed, by replacing *q* by $q^4$, and letting $a=b=c=d=q^r$, we obtain from (5.1), after multiplying both sides by [*r*], the following identity:

5.2 $$\begin{aligned} \sum _{k\ge 0}[8k+r]\frac{(q^r;q^4)_k^4}{(q^4;q^4)_k^4}q^{(4-2r)k}&=[r]\frac{(q^{4+r},q^{4-r},q^{4-r},q^{4-r};q^4)_\infty }{(q^4,q^4,q^4,q^{4-2r};q^4)_\infty }\nonumber \\&=\frac{[r]\,\Gamma _{q^4}(1-\frac{r}{2})}{\Gamma _{q^4}(1+\frac{r}{4})\Gamma _{q^4}(1-\frac{r}{4})^3}, \end{aligned}$$

valid for $r<2$. In the last equation we have rewritten the product using the *q*-Gamma function

$$\begin{aligned} \Gamma _q(x)=\frac{(q;q)_\infty }{(q^x;q)_\infty }(1-q)^{1-x}, \end{aligned}$$

defined for $0<q<1$ (cf. [4, Section 1.10]). For $r\le 1$ being an odd integer, we have thus just established an archimedian analogue of Theorem 6.

Now, since $\lim _{q\rightarrow 1^-}\Gamma _q(x)=\Gamma (x)$, we obtain that in the $q\rightarrow 1^-$ limit (5.2) becomes

5.3 $$\begin{aligned} \sum _{k\ge 0}(8k+r)\frac{(\frac{r}{4})_k^4}{k!^4} =\frac{r\,\Gamma (1-\frac{r}{2})}{\Gamma (1+\frac{r}{4})\Gamma (1-\frac{r}{4})^3} =\frac{4\,\sin (\frac{r\pi }{4})\,\Gamma (1-\frac{r}{2})}{\pi \Gamma (1-\frac{r}{4})^2}, \end{aligned}$$

where we have used the well-known reflection formula for the Gamma function. It is now immediate that for $r=1$ we get (1.1) while for $r=-1$ we get the similarly attractive evaluation

5.4 $$\begin{aligned} \sum _{k=0}^\infty (8k-1)\frac{(\frac{-1}{4})_k^4}{k!^4} =\frac{-16\sqrt{2}}{\sqrt{\pi }\,\Gamma (\frac{1}{4})^2}. \end{aligned}$$

Many other identities involving $\pi$ can similarly be obtained. At this place, in passing, we would like to point out that by replacing *q* by $q^2$ in (5.1) and putting $a=q^2$, $b=c=d=q$ one readily obtains

5.5 $$\begin{aligned} \sum _{k\ge 0}\frac{1+q^{2k+1}}{1+q}\frac{(1-q)^2}{(1-q^{2k+1})^2}q^k =\frac{(q^4,q^2,q^2,q^2;q^2)_\infty }{(q^3,q^3,q^3,q;q^2)_\infty }, \end{aligned}$$

which, as recently noted by Sun [29, Equation (1.3)] (who derived this identity by completely different means) is easily seen to be a *q*-analogue of Euler’s identity

$$\begin{aligned} \sum _{k\ge 0}\frac{1}{(2k+1)^2}=\frac{\pi ^2}{8}, \end{aligned}$$

used to prove his famous evaluation $\zeta (2)=\pi ^2/6$. See [34] for recent new samples of expansions involving $\pi$, obtained by suitably specializing *q*-series identities.

We turn to discussing whether some of the results obtained in the paper can be further strengthened. We have proved Theorems 2 and 3 by establishing a common generalization of them, namely Theorem 7. However, we are unable to prove a similar common generalization of (1.3) and (1.4). Numerical calculation for $q=1$ suggests that there are no congruences for the left-hand side of (3.4) with *odd* $d\geqslant 5$ that would hold in general (in particular, the case $d=5$ and $r=3$ appears to be such a counterexample).

Nevertheless, we would like to give the following result being similar to Theorem 7.

### Theorem 8

Let $d\geqslant 5$ be an odd integer and let *r* be an even integer with $\gcd (d,r)=1$. Let $n>1$ be an odd integer with $n\equiv -r\pmod {d}$ and $n\geqslant \max \{r,d-r\}$. Then

5.6 $$\begin{aligned} \sum _{k=0}^{n-1}[2dk+r]_{q^2}\frac{(q^{2r};q^{2d})_k^d}{(q^{2d};q^{2d})_k^d} q^{d(d-r-2)k} \equiv 0\pmod {\Phi _n(q)^2}. \end{aligned}$$

The proof of Theorem 8 is similar to that of Theorem 7. In this case we need to apply Andrews’ transformation (3.5) with $m=(d-1)/2$, $q\rightarrow q^{2d}$, $a=q^{2r}$, $b_1=q^{d+r}$, $b_2=\cdots =b_{m}=q^{2r}$, $c_1=\cdots =c_{m-1}=q^{2r}$, $c_m={q^{2r+2(d-1)n}}$ and $N=((d-1)n-r)/d$. The details of the proof are omitted here.

We can also prove the following refinement of (1.4) and (1.8). However, we are unable to deduce any interesting conclusion similar to (1.10) from this result by letting $q\rightarrow 1$.

### Theorem 9

Let $d\geqslant 3$ be an integer and let $n>1$ be an integer with $n\equiv 1\pmod {d}$. Then

$$\begin{aligned} \sum _{k=0}^{n-1}[2dk-1]\frac{(q^{-1};q^d)_k^d}{(q^d;q^d)_k^d}q^{\frac{d(d-1)k}{2}} \equiv 0\pmod {\Phi _n(q)^2\Phi _{dn-n}(q)}. \end{aligned}$$

We would like to propose the following three conjectures which are similar to Corollary 5.

### Conjecture 1

Let *r* be a positive integer and let *p* be a prime with $p>2r+1$. Then

$$\begin{aligned} \sum _{k=0}^{p-1}k^r \left( k+\frac{1}{p+1}\right) ^r \frac{(\frac{1}{p+1})_k^{p+1}}{k!^{p+1}}\equiv 0\pmod {p^4}. \end{aligned}$$

### Conjecture 2

Let $p>3$ be a prime. Then

5.7 $$\begin{aligned} \sum _{k=0}^{p-1}(2pk+2k+1)\frac{(\frac{1}{p+1})_k^{2p+2}}{k!^{2p+2}} \equiv 0\pmod {p^7}. \end{aligned}$$

More generally, if $p>3$ is a prime and *r* a positive integer, then

5.8 $$\begin{aligned} \sum _{k=0}^{p^r-1}\Big (2k\frac{p^{r+1}-1}{p^r-1}+1\Big ) \frac{\big (\frac{p^r-1}{p^{r+1}-1}\big )_k^{2\frac{p^{r+1}-1}{p-1}}}{k!^{2\frac{p^{r+1}-1}{p-1}}} \equiv 0\pmod {p^{2r+5}}. \end{aligned}$$

### Conjecture 3

Let $p>3$ be a prime. Then

5.9 $$\begin{aligned} \sum _{k=0}^{p-1}(2pk-2k-1)\frac{(\frac{-1}{p-1})_k^{2p-2}}{k!^{2p-2}} \equiv 0\pmod {p^5}. \end{aligned}$$

More generally, if $p>3$ is a prime and *r* a positive integer, then

5.10 $$\begin{aligned} \sum _{k=0}^{p^r-1}(2kp^r-2k-1)\frac{(\frac{-1}{p^r-1})_k^{2p^r-2}}{k!^{2p^r-2}} \equiv 0\pmod {p^{2r+3}}. \end{aligned}$$

Conjectures 2 and 3 are quite remarkable as they concern supercongruences modulo high prime powers. We now give two partial *q*-analogues of (5.7) as follows.

### Conjecture 4

Let *n* be an integer greater than 1. Then

$$\begin{aligned} \sum _{k=0}^{n-1} [2nk+2k+1]\frac{(q;q^{n+1})_k^{2n+2}}{(q^{n+1};q^{n+1})_k^{2n+2}} q^{(n+1)(n-1)k}\equiv 0\pmod {[n]^2\Phi _n(q)^2\Phi _{n^2}(q)}. \end{aligned}$$

### Conjecture 5

Let *p* be a prime. Then

5.11 $$\begin{aligned}&\sum _{k=0}^{p-1} [2pk+2k+1]\frac{(q;q^{p+1})_k^{2p+2}}{(q^{p+1};q^{p+1})_k^{2p+2}} q^{(p+1)(p-1)k}\nonumber \\&\quad \equiv -\frac{(2p+1)(p+1)^2p(p-1)}{72}q(1-q)^2[p]^4\Phi _{p^2}(q) \pmod {[p]^5\Phi _{p^2}(q)}. \end{aligned}$$

It is clear that the $q\rightarrow 1$ case of (5.11) reduces to (5.7) modulo $p^6$. We would like to emphasize that (5.11), while still conjectural, appears to be the first example of a basic hypergeometric supercongruence in the existing literature, that in the limit $q\rightarrow 1$ reduces to a supercongruence (for a hypergeometric series being truncated after a number of terms that is linear in *p*) modulo $p^6$.

Finally, we give a partial and a complete *q*-analogue of (5.9) as follows.

### Conjecture 6

Let *n* be an integer greater than 1. Then

$$\begin{aligned} \sum _{k=0}^{n-1} [2nk-2k-1]\frac{(q^{-1};q^{n-1})_k^{2n-2}}{(q^{n-1};q^{n-1})_k^{2n-2}} q^{(n-1)^2k}\equiv 0\pmod {[n]^2\Phi _n(q)^2}. \end{aligned}$$

### Conjecture 7

Let *p* be a prime. Then

5.12 $$\begin{aligned}&\sum _{k=0}^{p-1} [2pk-2k-1]\frac{(q^{-1};q^{p-1})_k^{2p-2}}{(q^{p-1};q^{p-1})_k^{2p-2}} q^{(p-1)^2k}\nonumber \\&\quad \equiv \frac{(2p-3)(p-1)(p-2)^2(p-3)}{6}(1-q)^2[p]^4 \pmod {[p]^5}. \end{aligned}$$

It is clear that the $q\rightarrow 1$ case of (5.12) reduces to the modulo $p^5$ congruence in (5.9).
